# Enduring deficits in memory and neuronal pathology after blast-induced traumatic brain injury

**DOI:** 10.1038/srep15075

**Published:** 2015-11-05

**Authors:** Venkata Siva Sai Sujith Sajja, W. Brad Hubbard, Christina S. Hall, Farhad Ghoddoussi, Matthew P. Galloway, Pamela J. VandeVord

**Affiliations:** 1 School of Biomedical Engineering and Sciences, Virginia Polytechnic and State University, Blacksburg, VA; 2 Department of Anesthesiology, Wayne State University School of Medicine, Detroit, MI; 3 Department of Psychiatry, Wayne State University School of Medicine, Detroit, MI; 4 Salem VA Medical Center, Research & Development Service, Salem, VA, USA

## Abstract

Few preclinical studies have assessed the long-term neuropathology and behavioral deficits after sustaining blast-induced neurotrauma (BINT). Previous studies have shown extensive astrogliosis and cell death at acute stages (<7 days) but the temporal response at a chronic stage has yet to be ascertained. Here, we used behavioral assays, immmunohistochemistry and neurochemistry in limbic areas such as the amygdala (Amy), Hippocampus (Hipp), nucleus accumbens (Nac), and prefrontal cortex (PFC), to determine the long-term effects of a single blast exposure. Behavioral results identified elevated avoidance behavior and decreased short-term memory at either one or three months after a single blast event. At three months after BINT, markers for neurodegeneration (FJB) and microglia activation (Iba-1) increased while index of mature neurons (NeuN) significantly decreased in all brain regions examined. Gliosis (GFAP) increased in all regions except the Nac but only PFC was positive for apoptosis (caspase-3). At three months, tau was selectively elevated in the PFC and Hipp whereas α-synuclein transiently increased in the Hipp at one month after blast exposure. The composite neurochemical measure, *myo*-inositol+glycine/creatine, was consistently increased in each brain region three months following blast. Overall, a single blast event resulted in enduring long-term effects on behavior and neuropathological sequelae.

Blast-induced neurotrauma (BINT) is a debilitating condition often affecting cognition. In recent years, the prevalence of BINT has increased due to military conflicts. In the US, as troops are returning from combat, the prevalence of soldiers with blast exposure correlating with psychological and psychiatric deficits has increased^1^,^2^. Exposure to the primary blast wave may not show visible diagnostic signs of injury but can cause significant neurological damage. Oxidative stress has been commonly associated with BINT as a critical factor affecting mitochondrial function and dysfunction in glucose metabolism^3^,^4^,^5^,^6^. In addition, studies have shown that impaired regulation of glucose metabolism can lead to Krebs cycle dysfunction and decreased ATP production resulting in cellular distress^3^,^7^,^8^. Furthermore, previous research has shown blood brain barrier (BBB) disruption and increased inflammatory markers, such as interferon (IFN)-γ, interleukin-1β (IL-1β) and glial fibrillary acidic protein (GFAP), following BINT^3^,^4^,^5^,^7^. The presence of neuroinflammation has been further supported by increased membrane turnover markers, such as glycerophosphocholine (GPC) and phosphorylethanolamine (PEA), leading to apoptosis in the hippocampus^3^. Cholinergic, dopamine and serotonin systems also have been shown to be affected following BINT^6^,^9^. This could alter signaling cascades dramatically, leading to apoptosis and altered neuronal communication across different regions of the brain.

It has been reported that regions of the brain, such as the amygdala, cerebellum, hippocampus, nucleus accumbens, and prefrontal cortex, display elevated levels of apoptosis at acute to sub-acute stages following BINT^3^,^4^,^5^,^8^,^10^,^11^,^12^. The majority of reports have shown loss of neuronal populations, while also showing an increase in astrogliosis^3^,^4^,^5^,^6^,^7^,^8^,^9^,^10^,^11^,^12^. The limited number of magnetic resonance imaging studies combined with histopathological data have shown the vulnerability of oligodendrocytes and endothelial cells following BINT^13^,^14^,^15^. In conjunction with molecular changes, behavioral abnormalities such as memory impairment and anxiety have been widely demonstrated in acute pre-clinical studies^11^,^16^,^17^. These changes align with the display of clinical symptoms following BINT. Although the response of BINT has been examined at acute stages (<5 days), the mechanism of cellular injury progression and biological markers is not completely understood in order to explain delayed clinical diagnosis of neurotrauma^3^,^4^. In addition, regions of the brain that play important roles in emotional stress and fear conditioning, such as the amygdala and nucleus accumbens, have been understudied. Few reports exist regarding the pathology of the amygdala and nucleus accumbens^9^,^16^,^17^. Furthermore, the lack of in-depth understanding regarding the pathology of the prefrontal cortex could further limit the knowledge surrounding loss of memory due to BINT. Connections between BINT and post-traumatic stress disorder (PTSD) have been established in the context of clinical landmarks^18^. BINT has been shown to cause a display of clinical symptoms such as short-term memory loss and learning^19^,^20^. Few pre-clinical reports have suggested that BINT can lead to up-regulation of dementia associated markers^7^,^12^. It is critical to recognize the role of biochemical markers such as tau and prion proteins, which are associated with dementia, in the hippocampus and prefrontal cortex to understand the pathological underpinnings of BINT^7^,^12^. In addition, understanding the long-term consequences of BINT on the amygdala, hippocampus, nucleus accumbens, and prefrontal cortex could potentially open a window for therapeutic interventions and diagnosis.

Characterizing the temporal progression of BINT is important for studying slow-developing symptoms that can be used for prognosis of military veterans who do not present with acute signs of this condition^19^. Studying the chronic effects in an experimental model of BINT allows for further discovery into the delayed effect on cellular injury cascades. It has been shown that there are deficits in hippocampal-dependent learning and memory at one month in a mouse model, however, other regions of the brain that are vital for learning and memory are understudied^20^.

Studies have been developed to examine how traumatizing events such as a blast wave exposure, can contribute to alterations in memory function and emotional distress^3^,^14^,^16^. Thus, the hypothesis of this study is that BINT induces alterations in acute neurochemistry and neuropathology that creates chronic aberrant behavior and neuropathology. In accordance to the hypothesis, previous studies have found increased levels of *myo*-inositol + glycine (Ins + Gly)/creatine (Cre), Iba-1 and GFAP, markers of gliosis, in rodents displaying memory impairment^11^. In addition, neurodegeneration was supported by the evidence of increased Fluoro-Jade B and decreased NeuN levels using immunohistochemistry.

Further links between BINT and dementia/Alzheimer’s–like disease can be observed through finding changes in neuropathology relating to spatial and short-term memory loss. Altogether, this study establishes BINT as a risk factor for dementia with supporting behavioral and pathological evidence. One exposure to blast could result in enduring memory loss, anxiety, and accompanying pathology. In addition, changes that occur at an early time point could be used to predict long-term pathological changes following blast exposure.

## Results

### Anxiety and active avoidance

**Figure 1:**
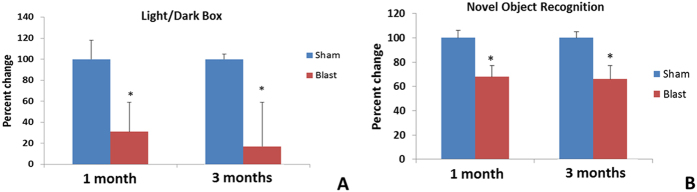
(**A**) Blast animals actively avoided the light chamber as evidenced by the significant decrease of latency time for initial entry into dark chamber (*p < 0.05). (**B**) Decreased time spent with the novel object suggested a long-term disruption of learning and memory in blast group (*p < 0.05). In each case, the response of the control group was assigned 100% and response of blast groups scaled accordingly.

Neuropathology within the amygdalar region due to brain injury has been shown to induce anxiety or avoidance in animals. Thus, light and dark box tests have been used to study behavior changes due to amygdalar pathology. Compared to the activity of controls (Fig. 1A), animals exposed previously to blast rapidly entered the dark chamber upon introduction to the apparatus (i.e. exhibited a decreased latency to enter the dark side, p < 0.05); however, there was no overall effect of blast exposure on the number of transitions or total time spent in either dark or light chambers.

### Novel Object Recognition

Primary blast injuries have been shown to affect memory in both clinical and pre-clinical studies with the hippocampus and prefrontal cortex being the primary effector regions. Memory retrieval and encoding is handled by neurotransmission between prefrontal cortex and hippocampus. Compared to the increased time that control animals spent with a novel object in the T2 phase of testing, the blast exposed animals (at both 1 and 3 months, p < 0.05) spent significantly less time with the novel object (Fig. 1B). Decreased time with a novel object under these experimental conditions is consistent with deficits in learning and/or short-term memory recall processes.

### Metabolic changes assessed using HRMAS ^1^H-MRS

Neurochemicals could be used as predictors of brain injury in terms of energy status, inflammation, oxidative stress and neurotransmission. This information is directly translatable as ^1^H-MRS can be used clinically. In conjunction with immunohistochemistry, more detailed pathological mechanisms could be understood in relation to behavior deficits.

### Amygdala (Amy)

At one month following blast exposure, there was significant increase in absolute concentrations of glutamate (Glu) (2.84 ± 0.11 nmol/mg vs 3.98 ± 0.56 nmol/mg) and cholines (sum of glycerophosphocholine (GPC), choline (Cho), phosphocholine (PCH)) (0.60 ± 0.11 nmol/mg vs 1.19 ± 0.26 nmol/mg) and a decrease in the ratio of γ-amino butyric acid (GABA)/creatine (Cre) (1.48 ± 0.18 vs 0.96 ± 0.12) and the ratio of GABA/Glu (0.80 ± 0.14 vs 0.49 ± 0.06) (p < 0.05). At three months following blast exposure, there was a significant increase in absolute concentrations of *myo*-inositol (Ins) (2.41 ± 0.41 nmol/mg vs 3.58 ± 0.22 nmol/mg), and the ratio of Ins + glycine (Gly)/Cre (2.04 ± 0.18 vs 3.27 ± 0.53) (p < 0.05).

### Hippocampus (Hipp)

At one month following blast exposure, a significant increase in absolute concentrations of Cho (0.14 ± 0.01 nmol/mg vs 0.31 ± 0.08 nmol/mg) was observed. At three months following blast exposure, a significant increase in the ratio of lactate (Lac)/Cre (2.21 ± 0.10 vs 2.63 ± 0.08), an increase in the ratio of Ins-Gly/Cre (1.65 ± 0.48 vs 2.89 ± 0.21) and a decrease in the ratio of glutamine (Gln)/Glu (0.58 ± 0.07 0.41 ± 0.03) (p < 0.05) was observed.

### Nucleus Accumbens (Nac)

At one month following blast exposure, there was significant increase in absolute concentrations of phosphorylethanol amine (Pea) (0.75 ± 0.12 nmol/mg vs 1.11 ± 0.07 nmol/mg) (p < 0.05). At three months following blast exposure, a significant increase was found in the ratios of Glu/Cre (1.68 ± 0.05 vs 1.84 ± 0.03), Ins/Cre (1.89 ± 0.17 vs 2.39 ± 0.07) and Ins-Gly/Cre (2.56 ± 0.34 vs 3.57 ± 030) (p < 0.05).

### Prefrontal cortex (PFC)

At one month following blast exposure, a significant increase in absolute concentrations of Glu (2.64 ± 0.41 nmol/mg vs 4.15 + 0.61 nmol/mg), Cho (0.06 ± 0.01 nmol/mg vs 0.13 ± 0.03 nmol/mg), Ins (1.29 ± 0.30 nmol/mg vs 2.26 + 0.33 nmol/mg), Ins-Gly (0.73 ± 0.31 nmol/mg vs 2.20 + 0.40 nmol/mg) and the ratio of Gln/Cre (0.49 ± 0.02 vs 0.57 ± 0.02) (p < 0.05) was observed. At three months following blast exposure, a significant decrease in the absolute concentrations of n-acetyl aspartate (NAA) (3.26 ± 0.18 nmol/mg vs 2.41 ± 0.27 nmol/mg) and cholines (1.02 ± 0.11 nmol/mg vs 0.41 ± 0.16 nmol/mg), and an increase in the ratio of Ins-Gly/Cre (1.54 ± 0.17 vs 2.56 ± 0.70) (p < 0.05) was observed.

### Immunohistochemistry

Markers for astrogliosis, cell death, and neurodegeneration were evaluated. A summary of all the raw data is presented in Table 1.

**Figure 2:**
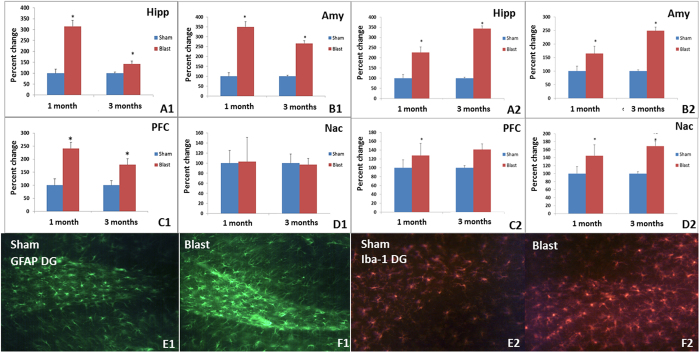
Left Panels: Increased astrogliosis (GFAP+) (*p < 0.05) was observed in Hipp (A1), Amy (B1) and PFC (C1), but no changes were observed in Nac (D1) when blast group was compared to control group at 1 and 3 months following exposure. Representative immunochemical images for GFAP in the dentate gyrus (DG) area of control (**E1**) and blast group (**F1**). Right panels: Increased microglia (Iba-1) (*p < 0.05) was observed in Hipp (**A2**), Amy (**B2**), PFC (**C2**) and Nac (**D2**) when blast group was compared to controls at **1** and 3 month following exposure. Representative immunohistochemistry images are shown for Iba-1 in the DG of control (**E2**) and blast groups (**F2**).

Astrogliosis: A significant increase in astrogliosis was observed with elevated levels of GFAP in blast group compared to control. Specifically, increased GFAP was found in the Amy, Hipp, and PFC regions but not the Nac region at one and three months. Iba-1 levels were increased significantly in Amy, Hipp, Nac and PFC regions at one and three months in the blast group compared to control (p < 0.05) (Fig. 2).

Cell death: Significant increase in caspase-3 was observed in PFC but not Amy, Hipp, or Nac at one and three months in blast compared to control group (p < 0.05) (Fig. 2).

**Figure 3:**
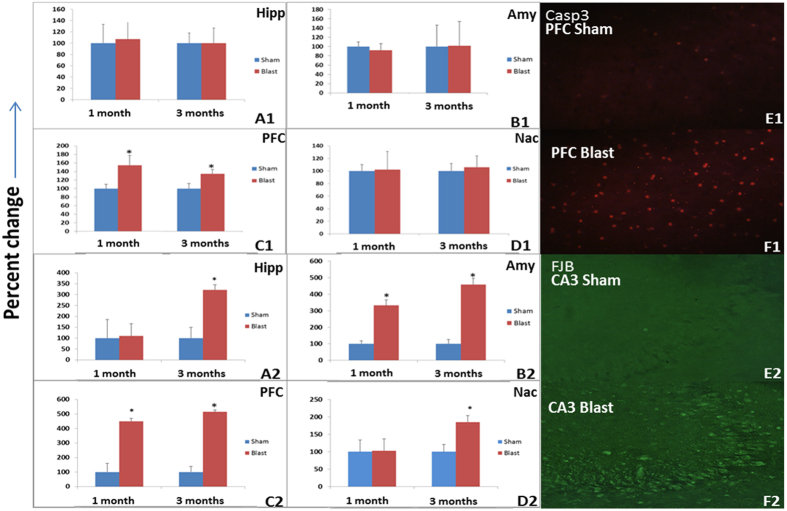
Top panels: Only the PFC (C1) showed signs of apoptotic activity in the extended period after blast. No significant increases in caspase-3 were noted in Hipp (**A1**), Amy (**B1**) or Nac (**D1**) when compared to controls at 1 month. Representative immunohistochemistry images for caspase-3 in PFC from control (E1) and blast groups (F1). Bottom panels: Increased neurodegeneration (FJB+ neurons) was observed in all regions at 3 months whereas only the Amy (**B2)** and PFC (**C2**) showed degeneration at one month (*p < 0.05). Representative immunohistochemistry images are shown for Fluoro-Jade B in CA3 of Hipp from control (**E2**) and blast groups (**F2**).

**Figure 4:**
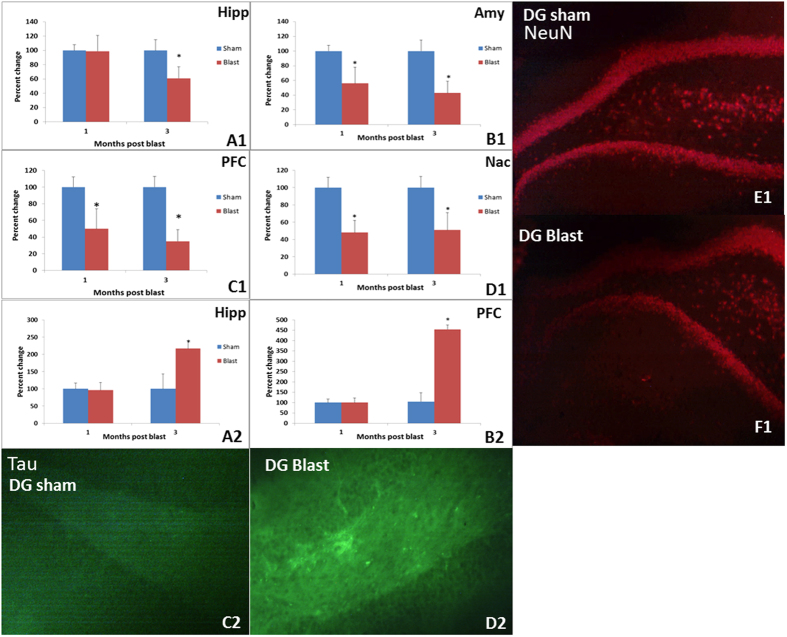
Top panels: Staining for NeuN, a protein found exclusively in mature neurons, was decreased in Amy (**B1**), Hipp (**A1**), Nac (**D1**), and PFC (**C1**) of blast animals (*p < 0.05). Representative immunochemical staining for NeuN (red) in the DG of control (**E1**) and blast groups (**F1**) are shown. Bottom panels: Increased tau pathology was observed in Hipp (**A2**) and PFC (**B2**) obtained three months after blast, when compared to control group (*p < 0.05). Representative immunochemical staining for tau (green) in DG from control (**C2**) and blast group animals at 3 months (**D2**) are shown.

Neurodegeneration and neuronal loss: Increased FJB immunoreactivity suggested neurodegeneration in Amy and PFC at both time points after blast whereas the Hipp and Nac showed degeneration only at three months (Fig. 3). The neuron-specific transcription factor NeuN decreased in Amy, Nac and PFC at both time points, whereas hippocampal NeuN was decreased only at the three month post-blast. (p < 0.05) (Fig. 3). Increased alpha-synuclein was observed in CA3 region of Hipp at one month post blast but no changes were observed in other regions of Amy, Hipp, or PFC at one and three months (Data not shown). Increases in tau protein aggregation were observed in Hipp and PFC at three months following blast exposure but not in Amy or Nac (Fig. 4).

## Discussion

Numerous pathological outcomes have been identified in relation to BINT. Neurochemical alterations/imbalance, oxidative stress, mitochondrial dysfunction and BBB disruption have been identified to be important pathological events that are involved at acute stages following BINT^3^,^4^,^5^,^6^,^7^,^8^,^9^,^10^,^11^,^12^,^13^,^14^,^15^,^21^,^22^,^23^. However, the long-term effects on various pathological markers and associated cognitive deficits are currently unclear. Assessing subfields of all the regions is an important future aim to further understand how the subfields contribute to the noted pathology of BINT. In previous studies, acute changes (3–48 hours) in neurochemistry were observed to be primarily associated with oxidative stress (reduced levels of glutathione or superoxide dismustase 1 (SOD1)) in Hipp, Nac and PFC regions following BINT^3^,^9^,^11^. Oxidative stress is known to initiate cell injury pathways eventually causing cell death. This process is primarily triggered by mitochondrial failure leading to compromised energy metabolism, inflammation due to cell membrane rupture, and irregular cellular homeostasis caused by the depletion of energy^24^.

Oxidative stress acts directly on mitochondrial processes binding to NAD/NADPH decreasing the production of ATP required for cellular homeostasis^24^,^25^. This harmful environment creates cellular stress which could translate into impaired signaling networks leading to long-term pathology. In this study, the primary cognitive regions of brain (Amy, Hipp, Nac and PFC) were observed to have a substantial loss of neurons and elevation in activated astrocytes. Chronic working memory issues and anxiety-associated behavior was related to chronic activation of astrocytes in Hipp and microglia in Amy respectively.

### Neurochemical assessment

Although acute studies depict energy level distress leading to cellular death, a sub-acute study of the PFC has shown a direct relationship between levels of Ins and impaired short term memory following BINT exposure^18^. In accordance to this study, increased levels of Ins + Gly could depict the progression of memory impairment in association with astrogliosis. Sub-acute data (3–7 days) depicted, ongoing metabolic stress by the increase in ratios of Glu/Gly or GABA/Glu. However, the conversion of these metabolites occurs in astrocytes as a supportive cascade for glutaminergic or GABAergic neurons in the brain^26^,^27^,^28^. This supports the idea that other cellular populations, such as oligodendrocytes and other glial cells, could be more sensitive to blast exposure but this hypothesis needs further evaluation. Increased levels of choline, a marker of inflammation, has been confirmed in Amy, Hipp, and PFC by cellular membrane turnover^3^,^29^. Alternatively, this could be representative of unbalance in the cholinergic system and the lack of homeostatic control could result in elevation of choline levels in these regions. In addition, elevated glutamate levels could be a contributing factor for excitotoxicity. Evidence of decreased NAA levels could also be associated with increased glutamate levels, as NAA buffers glutamate levels to form NAAG^30^. However, increased NAAG and overall decreased NAA could be an indicator of decreased neuronal population in PFC. Furthermore, changes in GABA/Glu and Gln/Glu in the Amy and Hipp, respectively, showed altered glutamergic neurotransmission. Glutamatergic neurotransmission plays a critical role in memory formation and mood-disorders^31^. This evidence was further supported by changes in Glu levels in the Nac and PFC.

Interestingly, elevated levels of Ins + Gly, a marker of astrogliosis, was found in all regions at a chronic stage of three months post blast^11^,^32^. Ins has been established to be an astrocyte marker and glycine is upregulated in astrocytes due to Glu breakdown^11^,^26^,^27^,^28^. Therefore, the combination of Ins + Gly can specifically relate to reactive astrogliosis. Clinically, increased Ins + Gly is being used to grade astrocytomas in glioma studies, which strengthens Ins + Gly as a marker of reactive astrocytes^26^,^32^. The ratio of Ins + Gly/Cre suggests that creatine, an abundant compound in the brain, could be unaltered possibly due to fact the small changes could be hard to identify using MRS.

Clinical studies have shown increased Gly levels in dementia and Alzheimer’s disease (AD) pathology using ^1^HMRS. Elevated Ins/Cre ratio has been associated with β-amyloid load using positron emission tomography (PET) in the elderly with normal cognition, and is typically elevated in mild cognitive impairment and AD dementia^33^,^34^. Thus, Ins + Gly could be a marker to identify prognosis of mild cognitive impairment following blast exposure^34^,^35^.

### Behavioral outcomes (working memory and anxiety) vs neuropathological deficits following blast exposure:

Behavioral deficits are reportedly due to the pathological sequelae of neurochemical changes, apoptosis and astrogliosis^3^,^9^,^11^. Several psychiatric and psychological disorders present themselves with neurochemical changes and specific pathology following brain injuries (TBI, stroke)^33^,^34^. Preclinical and clinical studies have shown persistent cognitive deficits and anxiety following blast exposure^5^,^11^,^16^,^17^,^36^. As such, these behavioral outcomes were assessed. Increased anxiety can result from the pathology of cells leading to neurodegeneration due to a continual biologically stressed environment. Biological stress was observed to be consistent in Nac with a loss neurons and intracellular oxidative stress resulting in astrogliosis.

Evidence of neuronal loss was supported by increased neurodegeneration and loss of neurons found at one and three months. The data suggested that neuronal loss leads to a marked increase of astrogliosis in Amy, Hipp, Nac and PFC. All regions evaluated were found to have an acute loss of neurons from the previous literature^3^,^9^,^21^,^37^. This loss of neurons was sustained throughout the three month evaluation period. The amount of neuronal population that was decreased was similar in all regions. In addition, an increase in tau protein aggregation suggested the tau tangles could be a contributing factor for neurodegeneration due to its toxicity. Previous reports from Huber *et al.* (2013) and Kochanek *et al.* (2013) support the evidence of dementia (esp. vascular dementia) and have shown increased tau and phosphorylated tau tangles up to one month following blast exposure^7^,^12^.

An increase of microglia, evaluated using Iba-1, suggested that microglia levels are increasing in number to assist with the injury repair process. In addition to the role of microglia in the inflammatory process, increased choline or changes in betaine, the precursor to choline, were found at one or three month in the four regions of the brain that were under investigation signifying ongoing inflammation^38^,^39^. Levels of α-synuclein, a presynaptic protein important for vesicle recycling, increased significantly in the CA3 region of the Hipp and the microtubule structural protein tau increased in the Hipp and PFC. Collectively, the results from neurochemical assessment demonstrated increases of Ins + Gly and Glu/Gly supporting the evidence of astrogliosis. These biological outcomes appear to be associated with the behavior deficits in learning, memory and active avoidance following BINT.

With the advancement of technology, clinical MRS (or NMR) could resolve peaks of Glu, GABA, Lac, Cre, Ins, cholines and NAA. NMR can be used as a critical diagnostic tool and measure of treatment effectiveness in BINT to understand the changes in the essential cognitive regions like Amy, Hipp, Nac, and PFC. However, the use of NMR for neuroscience is in a nascent stage as a diagnostic tool. Understanding the metabolic profiles clinically would provide insight into the potential changes which can be related to animal studies that identify key metabolic changes as well as for the identification of key biomarkers.

## Materials and Methods

### Animals and blast methodology

All the experiments are in accordance with The Virginia Tech Institutional Animal Care and Use Committee and all the experimental protocols described herein have been approved. Prior to all experiments, male Sprague Dawley rats (~250 g, Harlan Labs, San Diego) were acclimated to a 12 hour light/dark cycle with food and water provided ad lib. As described previously, the shock front and dynamic overpressure were generated using a custom-built Advanced Blast Simulator (ABS) (200 cm × 30.48 cm × 30.48 cm) that consists of a driving compression chamber attached to a rectangular transition and testing chamber with an end wave eliminator (ORA Inc. Fredericksburg, VA) located at the Center for Injury Biomechanics of Virginia Tech University. A passive end-wave eliminator (EWE) was installed at the venting end of the ABS, which minimizes the shock wave outflow by means of a specially designed plate system. Patterns in the EWE plate system were created to mirror reflected shocks and rarefactions, which tend to ‘cancel’ each other and diminish unwanted effects within the test section. A peak static overpressure was produced with compressed helium and calibrated acetate sheets (Grafix Plastics, Cleveland, OH)^36^,^40^.

Pressure measurements were collected at 250 kHz using a Dash 8HF data acquisition system (Astro-Med, Inc, West Warwick, RI) and peak overpressures were calculated by determining wave speed (m/s) at the specimen position. A mesh sling was used to hold the animal during the exposure that allowed for minimal hindrance of the wave through the chamber and shock wave profiles were verified to maintain consistent exposure pressures between subjects. The animals were anesthetized with 3% isoflurane before being placed in a rostral cephalic orientation towards the shock wave. Whole body exposure is considered “on-axis” with the animal facing rostral cephalic orientation towards the blast. This exposure has minimal effect on the lungs of the animals, as the shock streamlines around the body. Thus, resulting exposure in this study creates a relatively specific brain injury and minimal poly-organ trauma. Animals were randomly separated into four groups based on time points (n = 12/group). Two groups were euthanized one month following blast or control. The additional two groups with euthanized at three months following blast or control. Blast groups were exposed to a single incident pressure profile resembling a ‘free-field’ blast exposure, single Friedlander-like waveform, that is in mild-moderate range at 17 psi (117 kPa) with a positive duration of 2.5 ms, while the control groups underwent the same procedures with the exception of blast exposure^36^,^40^. Housing conditions during recovery were identical to pretreatment and similar for all experimental groups. From each experimental group of N = 12/group for behavioral testing, six were randomly assigned to neurochemical assessments *ex vivo (*N = 6/group) and six for immunohistochemical analyses (N = 6/group).

### Light and Dark Box Test (L/D)

Animals were assessed for behavioral deficits at one and three months following blast. Locomotor activity in the light/dark (L/D) box is a benzodiazepine-responsive ethological measure of the conflict between the inherent motivation to explore a novel environment and the fear-avoidance of a perceived danger in the light^36^,^41^,^46^. In the present experiment, we used initial time-to-enter the dark side as an index of the state-level of anxiety and considered a decreased latency (from experiment start) to enter the dark as an enhanced anxiety-like, avoidance behavioral phenotype. The apparatus consisted of two acrylic compartments, one dark side closed with a lid and one light side. The measurements of the L/D box are 72 × 30.5 × 33.5 cm with the dark side equal to 35.5 × 30.5 × 33.5 cm and the light side 35.5 × 30.5 × 33.5 cm. Each rat was tested by placing it in the light area facing away from the dark compartment and was allowed to explore the novel environment for five minutes. To minimize environmental bias, the animal was left alone in the testing room for the duration of the exploratory time and the behavior measurements. Latency from experiment start to enter the dark chamber, light-dark transitions, and time spent on the light side were measured with Ethovision™ video tracking software (Noldus Information Technology, Leesburg, VA) as previously reported^36^. Whole-body position was used to determine compartment transitions in terms of calculating time spent in the light compartment, transitions, and initial latency.

### Novel Object Recognition Test (NOR)

The NOR test was used to measure short-term memory, specifically object recognition (5). Animals were tested at one or three months following blast exposure. Testing occurred in three phases including: acclimation = acclimate to novel environment, trial 1 (T1) = presentation of two similar objects, and trial 2 (T2) = presentation of a novel and familiar object. In the first phase, rats were acclimated to a custom-made open field testing chamber (79 × 79 × 35 cm) with dim lighting by allowing them to explore the empty chamber for five minutes (time used in all phases) for two consecutive days prior to testing as described previously^5^,^11^. The testing chamber was located in a closed room and behavior was digitally recorded with a camera located above the chamber and linked to a computer outside the room. After placing an animal in the chamber, the experimenter exited the room and viewed the animal on the computer linked to the camera. Automated tracking and scoring was verified by a rater blind to the treatment conditions. The testing chamber was cleaned with 70% ethanol in water between each use^5^,^11^.

Fraction of time spent at novel position = Time of animal spent at novel object location/(Time spent at novel object location ± familiar object location).

### High resolution magic angle spinning proton-magnetic resonance spectroscopy (HRMAS ^1^H-MRS) analysis

^1^H-MRS analysis was performed as described previously using a HRMAS modified technique in a 11.7 T magnetic field^3^,^9^,^11^. After behavioral assessments were completed, animals (N = 6/group) were euthanized, brains excised, placed into a chilled brain matrix, and then cut into 2 mm coronal slices. Slices were immediately frozen on solid CO_2_ and then contralateral, 1.5 mm diameter punches were taken from Amy, Hipp, Nac and PFC according to the Paxinos & Watson brain atlas^42^. Tissues were stored at −80 °C until ^1^H MRS neurochemical analysis. Detailed information on the neurochemical visibility on the ^1^H MRS spectra after blast has been provided in Sajja *et al.*, 2012^3^.

Frozen intact tissue samples were weighed (2–3 mg) then placed into a Bruker zirconium rotor (2.9 mm diameter, 10 μL capacity) containing 2.5 μL PO_4_ buffer (pH = 7.4), formate, NaN_3_, 3-(trimethylsilyl)-proprionic acid (TSP) and 2.5 μL of D_2_O. TSP served as an internal chemical shift reference (0.00 ppm), formate (8.44 ppm) for auto-phasing, and D_2_O to lock on the center frequency. Once prepared, the rotor was promptly placed into a Bruker 11.7T Avance 500 MHz spectrometer maintained at 4 °C and spun around its axis at 4.2 kHz; the spatial orientation of the rotor was 54.7° (the magic angle) relative to the longitudinal (or main) magnetic field (B_o_). Field inhomogeneities were adjusted using a semi-automatic shimming procedure (Bruker). Tissue spectra were acquired with a CPMG rotor-synchronized pulse sequence^43^.

Each spectrum was analyzed using LCModel software utilizing a linear combination of a custom set of 27 neurochemical model spectra (basis set) to fit known MR-visible neurochemicals and calculate the absolute concentration values for each neurochemical. The goodness of fit for each compound was determined with Cramer–Rao bounds <15% being required for further analysis^44^,^45^. Absolute concentrations of MR visible metabolites were corrected for tissue weight and were expressed as nmol/mg tissue weight.

### Immunohistochemistry analysis

Animals (N = 6/group) were anesthetized with isoflurane (3%), perfused transcardially with 4% paraformaldehyde and then extracted brains were fixed in 30% sucrose solution prior to sectioning. Fixed brains were embedded within optimal cutting temperature compound (Sakura Finetek USA, Inc., Torrance, CA) then frozen on solid CO_2_. Regions of interest (Amy, Hipp, Nac and PFC were prepared with a microtome at −20 °C. For Hipp, subfields CA1, CA2, CA3 and dentate gyrus were evaluated. Amy was not sub-regioned and reported as the whole amygdala.

Immunohistochemistry was performed on sections from regions of interest for glial fibrillary acidic protein (GFAP; an astrocyte specific cell activation indicator), ionized calcium-binding adaptor molecule 1(Iba-1; microglial marker), cleaved caspase-3 (apoptosis), neuronal nuclei staining (Neu-N; neuronal marker), α-synuclein (α-syn) and tau protein staining for pathology associated with memory impairment.

As described previously^36^, processed tissue sections were rinsed with phosphate buffered saline (PBS) and incubated in 0.5% Triton X-100 + 0.5% gelatin blocking buffer for one hour. After being washed with PBS, nonspecific binding sites were blocked with 3% bovine serum albumin (BSA) in PBS for one hour at room temperature. Sections were then incubated with a primary antibody anti-GFAP (Invitrogen, Carlsbad, CA), anti-Iba-1 (Biocare Medical, Concord, CA), anti-cleaved caspase-3 (Invitrogen, Carlsbad, CA)), anti-Neu-N (Millipore, Billerica, MA), anti- α-syn (Abcam, Cambridge, MA), anti-tau-5 (Invitrogen, Carlsbad, CA) overnight at 4 °C. After a PBS wash, the samples were incubated for one and a half hours with fluorescence-tagged fluorescein isothiocyanate (FITC)-secondary anti-rat IgG antibodies (1:250; Vector Laboratories, Burlingame, CA) or Alexa flour-555 anti-rabbit IgG antibody (1:250; Cell Signaling, Danvers, MA) for the respective antibody targets. After a PBS wash, samples were mounted on slides, air dried, and cover-slipped with prolong anti-fade gold reagent with 6-diamidino-2-phenylindole (DAPI; Invitrogen, Carlsbad, CA). Sections were examined under a Zeiss fluorescence microscope at 20× magnification under appropriate fluorescent filters^18^. Fluorescence intensity of the acquired digital images was quantified by ImageJ software (NIH, Bethesda, MD).

### Fluoro-Jade B analysis

Brain slices were incubated in 1% NaOH-80% ethanol, hydrated in 70% ethanol, and washed in distilled water. The sections were subsequently incubated at room temperature in 0.006% potassium permanganate (Sigma-Aldrich, St. Louis MO) on a rotating stage, rinsed in distilled water, and incubated in a 0.0004% solution of FJB (Histochem Inc., Jefferson, AR). All solutions were made in dH_2_O. The brain sections were then rinsed in distilled water, air-dried and placed on a slide warmer until fully dry. The dry slides were cleared in xylene and mounted with 1,3-diethyl-phenylxanthine (Sigma-Aldrich; St. Louis, MO). An observer blind to the experimental conditions carried out cell counting. Counts were based on the morphology, fluorescent intensity, size and location of specific neurons using a Zeiss epifluorescence microscope^18^. The number of FJB + fluorescence intensity of acquired digital images was quantified by ImageJ software (NIH, Bethesda, MD).

### Statistics

Effects of blast exposure were measured in separate experiments (i.e. one and three months following blast overpressure exposure). Immunohistochemistry studies were treated as two separate experiments to avoid unknown influences and were assessed using two-tailed analysis of variance (ANOVA) with p < 0.05 considered significant. A two-factorial repeated measured ANOVA with Bonferroni post-hoc test was used for the behavioral testing with p < 0.05 considered statistically significant. Significance was assessed using SPSS™ statistical software and p < 0.05 was considered statistically significant. Unless indicated otherwise, data are presented as mean ± standard error of the mean (SEM).

## Additional Information

**How to cite this article**: Sajja, V. S. S. S. *et al.* Enduring deficits in memory and neuronal pathology after blast-induced traumatic brain injury. *Sci. Rep.* **5**, 15075; doi: 10.1038/srep15075 (2015).

## Figures and Tables

**Table 1:** Consistent across brain regions, 3 months after blast, markers for neurodegeneration (FJB) and microglia activation (Iba-1) increased whereas an index of mature neurons (NeuN) decreased.

Time point →	One month (Mean ± SEM) Control vs Blast (*p < 0.05)	Three months (Mean ± SEM) Control vs Blast (*p < 0.05)
Type of Stain	Region of brain (integrated density)	Region of brain (integrated density)
GFAP Astrocyte specific protein, marker of astrogliosis	HIPP: 4752 ± 854 vs 14954 ± 4098*	HIPP: 4241 ± 206 vs 6074 + 766*
	AMY: 2982 ± 1173 vs 10143 ± 1170*	AMY: 1711 ± 749 vs 4557 ± 1008*
	PFC: 458 ± 113 vs 1103 ± 258*	PFC: 2467 ± 158 vs 4256 ± 214*
	NAC: 249 + 57 vs 251 ± 124	NAC: 435 ± 67 vs 426 ± 51
FJB Marker of neurodegeneration	HIPP: 5264 ± 947 vs 5786 ± 824	HIPP: 7249 ± 3532 vs 23376 ± 5565*
	AMY: 3573 ± 884 vs 11862 ± 1252*	AMY: 7405 ± 1489 vs 32407 ± 2371*
	PFC: 432 ± 258 vs 1944 ± 412*	PFC: 1668 ± 658 vs 8594 ± 1205*
	NAC: 432 ± 147 vs 447 ± 151	NAC: 697 ± 147 vs 1296 ± 251*
NeuN Neuron specific protein (Rbfox3)	HIPP: 1431 ± 113 vs 1415 ± 307	HIPP: 2022 ± 305 vs 1230 ± 192*
	AMY: 389 ± 61 vs 218 ± 46*	AMY: 328 ± 53 vs 147 ± 30*
	PFC: 1882 ± 230 vs 944 ± 226*	PFC: 1561 ± 195 vs 545 ± 3 7*
	NAC: 1116 ± 207 vs 535 ± 74*	NAC: 605 ± 76 vs 308 ± 61*
Tau Microtubule structure	HIPP: 5312 ± 1039 vs 5666 ± 1065	HIPP: 880 ± 190 vs 3995 ± 1337*
	PFC: 1071 ± 267 vs 1080 ± 144	PFC: 949 ± 600 vs 2061 ± 705*
Iba-1 Microglia specific calcium binding protein, microgliosis	HIPP: 7818 ± 444 vs 11775 ± 2019*	HIPP: 1647 ± 699 vs 5668 ± 1581*
	AMY: 4170 ± 256 vs 6864 ± 939*	AMY: 1225 ± 321 vs 3056 ± 531*
	PFC: 2532 ± 128 vs 3291 + 245*	PFC: 948 + 117 vs 1365 ± 102*
	NAC: 1123 ± 97 vs 1605 ± 101*	NAC: 1812 ± 258 vs 3044 ± 154*
Cleaved caspase-3 Protease required for apoptosis	HIPP: 1564 ± 523 vs 1678 ± 626	HIPP: 4623 ± 190 vs 4645 ± 159
	AMY: 1541 ± 153 vs 1419 ± 196	AMY: 6960 ± 316 vs 7133 ± 369
	PFC: 576 ± 58 vs 894 ± 205*	PFC: 1982 ± 131 vs 2854 ± 163*
	NAC: 175 ± 47 vs 178 ± 51	NAC: 375 ± 67 vs 406 ± 36

Similarly, gliosis (GFAP) increased at both time points in all regions except the Nac which showed no indication of scarring. Only the PFC was positive for apoptosis (caspase-3). Three months after insult, the microtubule protein tau was selectively elevated only in the Hipp and PFC, whereas α-synuclein transiently increased in the Hipp one month after blast exposure.
